# The Role of the Bile Microbiome in Common Bile Duct Stone Development

**DOI:** 10.3390/biomedicines11082124

**Published:** 2023-07-27

**Authors:** Jungnam Lee, Hye Jung Jeong, Hanul Kim, Jin-Seok Park

**Affiliations:** Digestive Disease Center, Department of Internal Medicine, Inha University College of Medicine, Inha University Hospital, Incheon 22332, Republic of Korea; gallary801105@gmail.com (J.L.); jhj4358@naver.com (H.J.J.); khu0830@gmail.com (H.K.)

**Keywords:** CBD stone, microbiome, metabolites, *Enterococcaceae*, *Enterococcus*, 16S rRNA gene sequencing

## Abstract

Introduction: Common bile duct (CBD) stones are a health concern for 10–20% of individuals with symptomatic gallstones, leading to health complications and placing a burden on healthcare systems. This study was initiated to investigate the changes in microbiome compositions and the metabolic signature associated with CBD stones. The research approach integrated taxonomic and functional data with metabolomics data, complemented by in vivo experiments. Methods: In a single tertiary healthcare institution, a total of 25 patients were enrolled who had undergone endoscopic retrograde cholangiopancreatography (ERCP) between February 2019 and January 2021. We harvested DNA from bile samples acquired from these individuals. The amplification of the bacterial 16S rRNA gene V3-V4 region was conducted through polymerase chain reaction (PCR), followed by sequencing. We utilized QIIME2 for a comprehensive data analysis. Furthermore, we performed a metabolomic analysis of the bile samples using nuclear magnetic resonance (NMR) spectroscopy. For the assessment of functional gene enrichment, we employed MetaboAnalyst 5.0. Lastly, we executed in vivo experiments on C57BL/6 mice and undertook histological examinations of tissue samples. Results: Out of the 25 study subjects, 17 underwent ERCP due to CBD stones (the CBD stone group), while the remaining 8 had the procedure for different reasons (the non-CBD stone group). An alpha diversity analysis showed a significantly greater microbial diversity in the bile samples of the non-CBD stone group (*p* < 0.01), and a beta diversity analysis confirmed the greater microbial compositional abundance in the gut microbiomes in this group (*p* = 0.01). A taxonomic analysis revealed that the abundances of *Enterococcaceae* and *Enterococcus* were higher in the bile microbiomes of the CBD stone group. A metabolic profile analysis showed that the acetate, formate, and asparagine levels were higher in the CBD stone group. A pathway enrichment analysis showed the metabolic pathways (Arginine and Proline Metabolism, Aspartate Metabolism, Glycine, and Serine Metabolism, and Ammonia Recycling pathways) that were associated with these differences. Preclinical experiments demonstrated systemic inflammation and extracellular trap formation in the CBD stone group. Conclusions: Our study highlights the importance of biliary dysbiosis and bile metabolites, specifically acetate and formate, in CBD stone development and progression. These findings have implications for the development of diagnostic and therapeutic strategies using microbiomes for CBD stones.

## 1. Introduction

Common bile duct (CBD) stones are common and affect 10–20% of individuals with symptomatic gallstones [1,2], and can cause a range of health problems, such as pain, jaundice, infection, and acute pancreatitis, imposing significant burdens on healthcare systems [1]. Endoscopic retrograde cholangiopancreatography (ERCP) is a commonly used minimally invasive procedure for treating CBD stones. However, recurrence has been reported in up to 20% of patients after ERCP, which highlights the need to better understand the pathogenesis of this disease.

The pathogenesis of CBD stones remains unclear, despite significant advances in diagnostic and therapeutic modalities. Recent studies have suggested that the intestinal microbiome plays a critical role in the pathogeneses of biliary tract diseases, including CBD stones. However, the relationship between the biliary microbiome and CBD stones has not been well characterized. The human biliary system has traditionally been considered to be sterile, but emerging evidence, including our previous findings, suggests that it harbors a complex microbiota that may play a role in the development of biliary tract diseases [3,4,5,6,7]. Although some studies have investigated the biliary tract microbiota in patients with cholangitis or biliary tract cancer, few have investigated the biliary microbiota of patients with CBD stones [8,9,10]. Furthermore, the metabolic consequences of the biliary microbiome in these patients remain largely unknown. The biliary tract microbiome is essential for maintaining the homeostasis of the liver and bile ducts, and alterations in its composition might result in the development of pathological conditions [11]. The implantation of bile duct microbiota not only affects the bile metabolism, but also causes local inflammation [12]. In addition, the microbiome in some species can cause chronic inflammation and lead to choledocholithiasis. However, whether there is a real association between the biliary microbiota, metabolites, and choledocholithiasis needs to be further confirmed [13,14,15]. Thus, knowledge of the specific microorganisms involved in CBD stone formation could aid the development of targeted therapies.

Bile metabolites are the byproducts of the various metabolic processes occurring within the liver and biliary tract and are essential for nutrient absorption and digestion. Alterations in bile metabolite profiles have been observed in numerous liver diseases, such as cirrhosis, cholestasis, and hepatocellular carcinoma [9,16,17]. Thus, in view of the potential impacts of bile metabolites on hepatobiliary health, it is essential that their roles in CBD stone development and the possible interconnections between the biliary microbiome and bile metabolites be understood [9,18].

In this study, we examined the potential pathogenic role played by the biliary microbiome in CBD stones and its metabolic consequences. To elucidate the contribution of the biliary microbiome and its metabolites to CBD stone pathogenesis, we conducted preclinical mouse experiments aimed at investigating the underlying mechanisms of this.

## 2. Methods

### 2.1. Analysis of the Bile Microbiomes of CBD Stone and Non-CBD Stone Patients

We conducted an extensive investigation of the bile fluid microbiomes of patients who underwent ERCP at our institution between February 2019 and January 2021. The eligible participants were adults aged ≥ 18 years old who required ERCP for bile duct decompression or had a confirmed diagnosis of CBD stones via computed tomography (CT) or magnetic resonance imaging (MRI). Patients who underwent ERCP for unrelated conditions, such as other biliary diseases, were also eligible and categorized into the non-CBD stone group. Patients with hemolytic anemia or severe liver disease were excluded, as were those with challenging endoscopic approaches due to esophageal stenosis, gastric outlet obstruction, or duodenal stenosis. In addition, patients with a medical history of thrombocytopenia or coagulopathy (PT-INR > 1.5; normal 0.85–1.25, platelet count < 60,000/mm^3^) were deemed ineligible.

### 2.2. Acquisition of Human Bile Fluid Samples

ERCP was performed using a standard side-viewing duodenoscope (TJF-260; Olympus Corporation, Tokyo, Japan) and a straight standard injection catheter. Following the successful completion of the therapeutic goals, an endoscopic nasobiliary drainage (ENBD) tube was positioned in the proximal CBD. To avoid possible contamination from the upper gastrointestinal tract, including the oral cavity, bile samples (20–30 cc) were collected over 24 h post-endoscopic interventions. The collected samples were promptly transferred to sterile sputum receptacles and stored at −80 °C until they were required for analysis.

### 2.3. DNA Extraction from Bile Samples

The bile samples were stored at −80 °C before being shipped to Bioeleven Co., Ltd. (Seoul, Republic of Korea) for DNA analysis and sequencing. Following centrifugation at 5000× *g* for 5 min at room temperature, the samples were resuspended in 500 µL of cetyltrimethylammonium bromide buffer, according to the manufacturer’s instructions. DNA extraction was performed using the Maxwell^®^ RSC PureFood GMO and Authentication Kit (Promega, Madison, WI, USA). The bacterial DNA concentrations were determined using a UV-vis spectrophotometer (NanoDrop 2000c; Thermo Fisher Scientific, Waltham, MA, USA) and the QuantiFluor^®^ ONE dsDNA System (Promega). All the DNA specimens were stored at −20 °C until they were required.

### 2.4. Polymerase Chain Reaction (PCR) Amplification of the Bacterial 16S rRNA Gene V3–V4 Region

The amplification of the V3-V4 variable region of the bacterial 16S rRNA gene via PCR was carried out using a two-step method and two primers (5′-TCGTCGGCAGCGTCAGATGTGTATAAGAGACAGCCTACG-GGNGGCWGCAG (forward) and 5′GTCTCGTGGGCTCGGAGATGTGTATAAGAGACA-GGACTACHVGGGTATC-TAATCC-3′ (reverse)). The resulting PCR products were subjected to 2% agarose gel electrophoresis and the 16S rRNA libraries were purified using magnetic beads (AMPure XP), according to the manufacturer’s instructions (Beckman Coulter, Wycombe, UK). The sample purity was confirmed using a Bioanalyzer 2100 (Agilent, Santa Clara, CA, USA). For second-round PCR, Illumina Nextera barcodes (Illumina, Inc., San Diego, CA, USA) were attached to first-step PCR products using i5 forward and i7 reverse primers. The amplified products were then purified as described for the first PCR round. The DNA was quantified using the QuantiFluor^®^ ONE dsDNA System (Promega), and the sample quality was confirmed using a Bioanalyzer 2100 (Agilent, Santa Clara, CA, USA). The 16S rRNA libraries and amplified genes were sequenced using a MiSeq v3 Reagent Kit (Illumina, Inc.).

### 2.5. Analysis of 16S rRNA Sequencing Data in Bile Fluid Samples

The 16S rRNA sequencing data were analyzed using the QIIME2 pipeline (version 2022.11) as follows. First, low-quality base reads with quality scores of <30 were filtered and trimmed using Trimmomatic v0.39. Second, the DNA sequences were clustered into amplicon sequence variants (ASVs) by reference-based clustering using the Greengenes rRNA database (release 138) [19]. Third, the species richness and microbial profile differences were determined using alpha and beta diversities calculated using QIIME2. The alpha diversities were calculated using the microbial diversities determined by assessing the ASV richness using three indices, namely the observed ASVs, Dominance D, and Shannon indexes. The beta diversities were used as a measure of the compositional dissimilarity between the ASVs on phylogenetic trees, and Bray–Curtis distance matrices were generated from the predicted metagenomes using QIIME and analyzed using ANOSIM (analysis of similarities).

### 2.6. Metabolomic Analysis by Nuclear Magnetic Resonance (NMR) Spectroscopy

The bile samples were pre-washed using 3 kDa spin filters (NANOSEP 3K, Pall Life Sciences, Ann Arbor, MI, USA) at 10,000 × gn for 2–3 h at 4 °C. The filtrates were mixed with NMR buffer (100 mM phosphate buffer in D2O (pH 7.3) and 1.0 mM 3-trimethylsilyl 2,2,3,3-d4 propionate (TMSP)) to a final volume of 600 μL and a final TMSP concentration of 0.5 mM. NMR was performed on 550 μL samples in 103.5 mm × 5 mm NMR tubes (Bruker). One and two-dimensional 1H-NOESY NMR data, including 1H-1H total correlation spectroscopy (TOCSY) and 1H-13C heteronuclear single quantum coherence (HSQC) data, were obtained using a Bruker Avance II 600 MHz spectrometer equipped with Topspin 3.6 software (Bruker Analytik, Rheinstetten, Germany). Chemical shifts were assigned using the Human Metabolome Database (HMDB) and Chenomx^®^ NMR Suite profiling software (Chenomx Inc. version 8.4). A total of 41 metabolites were quantified using Chenomx software, using TMSP as the internal standard. A statistical analysis was performed on the acquired data using R-studio and MetaboAnalyst 5.0.

### 2.7. Functional Gene Enrichment Analysis

A Functional Gene Enrichment Analysis was performed using the pathway enrichment module in MetaboAnalyst 5.0 (https://www.metaboanalyst.ca/, accessed on 27 April 2021) to investigate the metabolic pathway enrichments in the datasets. To reduce systematic bias and improve the data consistency, normalization was conducted using MetaboAnalyst 5.0. Features with >25% of values missing were removed, and the remaining missing values were replaced with the mean values of the original data. The datasets were then normalized using summation, mean-centered, and divided by the square roots of the variable standard deviations.

### 2.8. In Vivo Experiments

Eight-week-old male C57BL/6 mice were purchased from OrientBio (Seungnam, Republic of Korea) and housed in a temperature- (20–25 °C) and humidity (RH 40–45%)-controlled room under a 12 h light/dark cycle. The mice were acclimated for one week before use and provided with free access to a standard, non-purified rodent diet (Cargill Agri Purina, Inc., Seongnam, Republic of Korea) and water. After acclimation, all the mice were provided with a standard, non-purified diet and water containing 2.5% calcium L-lactate hydrate. The experiment was conducted with a total of 8 mice divided into two groups: the control group, consisting of 2 mice, and the treatment group, consisting of 6 mice. The treatment group received an administration of sodium acetate (25 mg) and sodium formate (3.04 mg) dissolved in 0.9% saline to achieve a total volume of 500 µL, which was administered intraperitoneally twice weekly until sacrifice. The mice in the treatment group were further divided into three subgroups for sacrifice. Two mice were sacrificed after 4 weeks and another two were sacrificed after 8 weeks. The remaining two mice had their dosages of sodium acetate and sodium formate increased to 50 mg and 6.08 mg, respectively, after the 4th week and were sacrificed at the end of the 17th week. All the mice were weighed three times weekly to monitor their progress. Harvested hepatobiliary organs were bisected, fixed in 10% formaldehyde, and then worked up using a general tissue-processing procedure. The tissue samples were stained with hematoxylin and eosin (H&E) and examined under a light microscope. Photographs were obtained using an AxioImager microscope (Carl Zeiss, Jena, Germany).

### 2.9. Statement on Ethics

The study protocol was approved by the Institutional Review Board of Inha University Hospital (INHAUH 2019-02-015). Written informed consent was obtained from all the human participants before the procedures, and the study was conducted in accordance with the ethical principles outlined in the Declaration of Helsinki. The preclinical study was approved and performed according to the guidelines issued by The Animal Care and Use Committee of Inha University (Approved number INHA 220421-818).

## 3. Results

### 3.1. Characteristics of the Study Subjects

Twenty-five individuals were included, and their bile fluids were collected and analyzed. Seventeen of the study subjects underwent ERCP for CBD stone management, while the remaining eight underwent ERCP for the following conditions: dilated bile duct (n = 4), a benign tumor (n = 1), benign biliary stricture (n = 1), CBD bile sludge (n = 1), and autoimmune pancreatitis (n = 1). The baseline characteristics of the CBD stone and non-CBD stone groups were similar and are presented in Table 1.

### 3.2. Alpha and Beta Diversities

**Figure 1:**
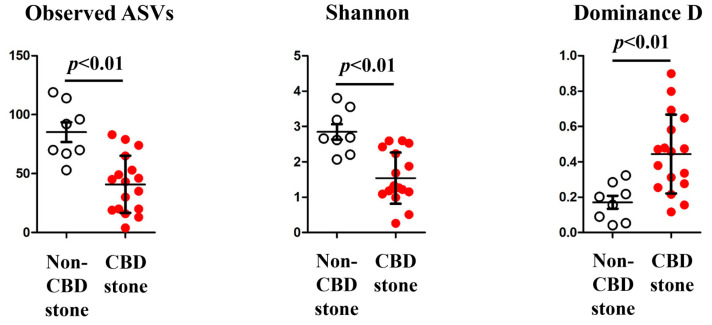
Alpha diversity (Observed ASVs, Shannon, and Dominance D). The observed ASVs, Shannon, and dominance D were compared between the non-CBD stone group (represented by white dots) and the CBD stone group (represented by red dots).

**Figure 2:**
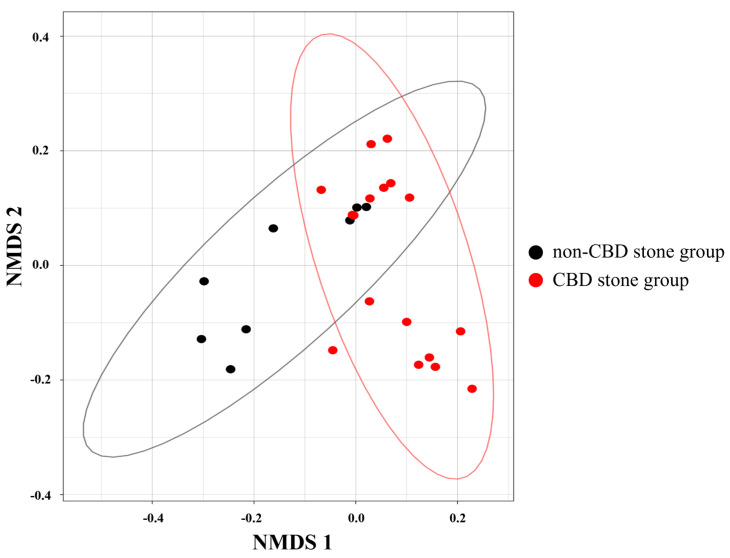
Beta diversity. Group beta diversity analysis results determined using Bray–Curtis distance matrices and the ANOSIM method.

The alpha diversity analysis revealed a significant dissimilarity in the microbial diversity between the bile fluids collected from the individuals in the CBD stone and non-CBD stone groups (Figure 1). Specifically, the observed ASVs (*p* < 0.01) and Shannon diversities (*p* < 0.01) were higher in the non-CBD stone group, indicating that the bile samples in this group contained more diverse microbial compositions. However, the Dominance D index (*p* < 0.01), which measures the extent to which one or several species prevail within a community, was higher in the CBD stone group. In the interpretation of these diversities, these results suggest that the bile samples of the CBD stone group exhibited less microbial diversity compared to those from the non-CBD stone group. Beta diversity, an important metric for assessing the differences in microbial compositions across groups, was used to further investigate the microbial profiles and also demonstrated a higher abundance of microbial compositions in the bile microbiomes of the non-CBD stone group (*p* = 0.01) (Figure 2). We also investigated the differences in the microbiome compositions based on age among the participating patients. There were no significant variations in the microbiome compositions based on age (Figure S1a,b).

### 3.3. Taxonomic Analysis of Bile Microbiomes

**Figure 3:**
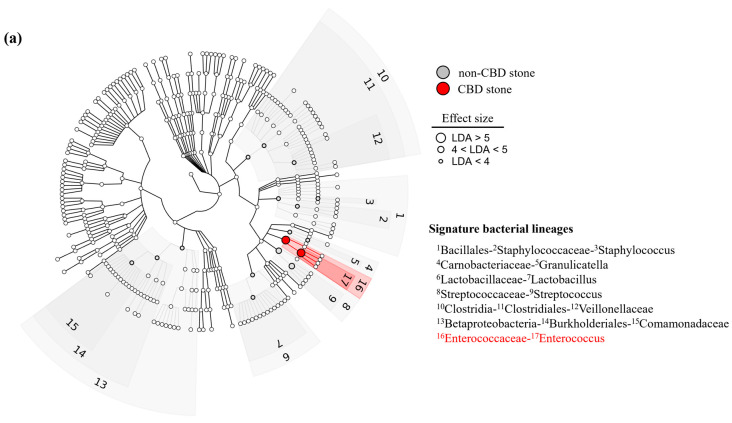

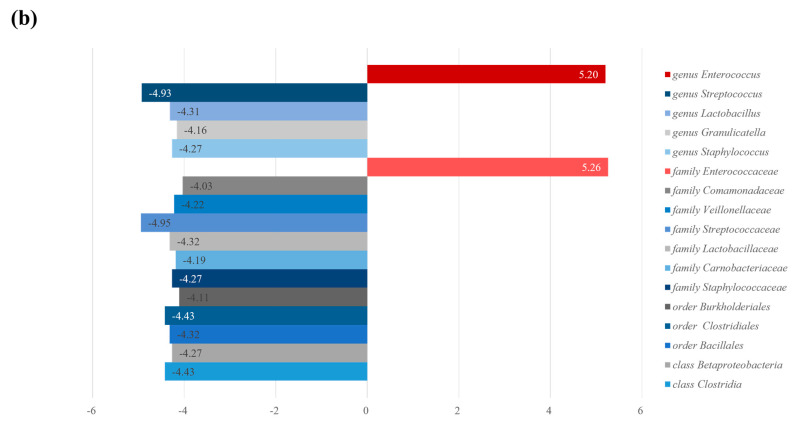
(**a**) Cladogram. Cladogram showing significantly different abundances of bacterial taxa between the CBD stone group and the non-CBD stone group. (**b**) LDA scores of bacterial taxa. LDA selection indicated a significant overrepresentation of the *Enterococcaceae* family (with an LDA score of 5.26) and the *Enterococcus* genus (with an LDA score of 5.20) in the group of patients with CBD stone group.

The linear discriminant analysis effect sizes (LEfSe) were determined to identify the major differences between the biliary microbiomes of the two groups. The linear discriminant analysis (LDA) selection showed the *Enterococcaceae* (LDA score 5.26) family and *Enterococcus* (LDA score 5.20) genus were significantly more abundant in the CBD stone group, whereas the *Clostridia* (LDA score −4.43) and *Betaproteobacteria* (LDA score −4.27) classes, the *Bacillales* (LDA score −4.32), *Clostridiales* (LDA score −4.43), and *Burkholderiales* (LDA score −4.11) orders, the *Staphylococcaceae* (LDA score −4.27), *Carnobacteriaceae* (LDA score −4.19), *Lactobacillaceae* (LDA score −4.32), *Streptococcaceae* (LDA score −4.95), *Veillonellaceae* (LDA score −4.22), and *Comamonadaceae* (LDA score −4.03) families, and the *Staphylococcus* (LDA score −4.27), *Granulicatella* (LDA score −4.16), *Lactobacillus* (LDA score −4.31), and *Streptococcus* (LDA score −4.93) genera were more enriched in the non-CBD stone group. Notably, the significance of the intergroup microbial compositional difference was primarily due to the bacteria of the phylum *Bacillota* (formerly *Firmicutes*). Most of the bacteria of this phylum were more abundant in the non-CBD stone group, except the bacteria of the *Enterococcaceae* family and the genus *Enterococcus*, which were more abundant in the CBD group. *Bacillota* (formerly *Firmicutes*) lineage bacteria were the key factor that differentiated between the two groups; the Enterococcus genus dominantly populated the bile of the CBD stone group, whereas more diverse *Bacillota* (formerly *Firmicutes*) bacteria (the *Clostridia* classes, the *Bacillales* and *Clostridiales* orders, the *Staphylococcaceae*, *Carnobacteriaceae*, *Lactobacillaceae*, *Streptococcaceae*, and *Veillonellaceae* families, and the *Staphylococcus*, *Granulicatella*, *Lactobacillus*, and *Streptococcus* genera) were significantly detected in the bile of the control group (Figure 3a,b). The compositions of the microbiomes at the major phylum and genus levels in the groups with and without CBD stones are depicted in Figure S2a,b.

### 3.4. Metabolic Profile Analysis of Bile Fluid

**Figure 4:**
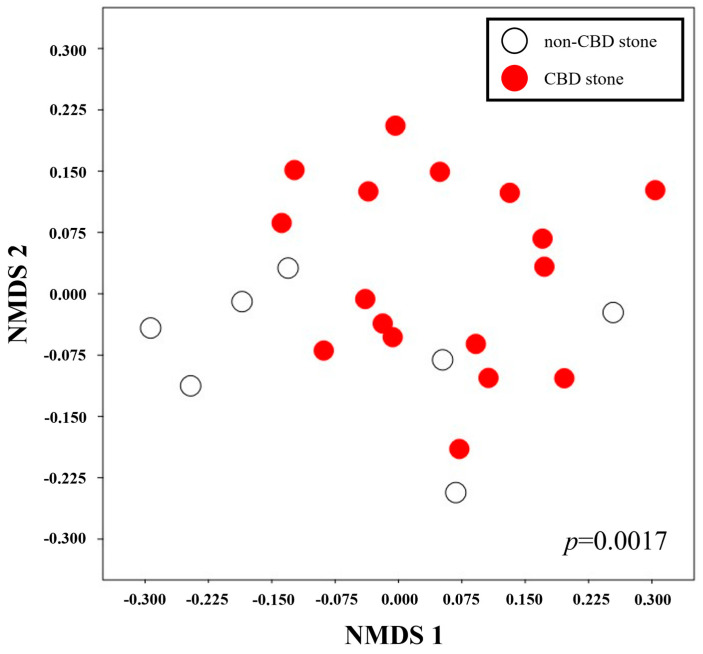
Analysis of similarity (ANOSIM). ANOSIM showed a significant difference between the metabolic profiles of bile samples from patients with or without CBD stones.

**Figure 5:**
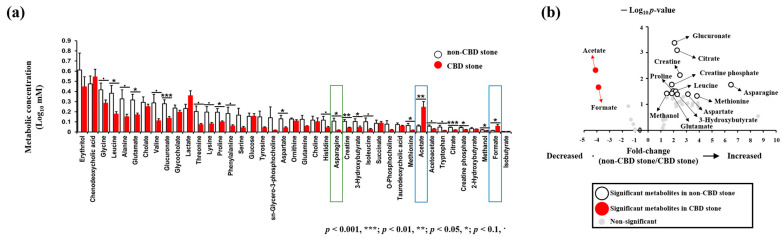
Signature metabolites. (**a**) Metabolite concentration analysis revealed elevated acetate and formate (blue boxes) levels in the CBD stone group, while the non-CBD stone group showed higher asparagine (green box) levels. (**b**) The metabolic concentrations were represented as fold-changes (non-CBD stone group/CBD stone group), where acetate and formate were significantly elevated in the CBD stone group compared to the non-CBD stone group.

To investigate the hypothesis that different metabolite profiles in the bile of CBD stone patients are caused by different microbial communities, we used NMR to identify these metabolic profiles. A proton nuclear magnetic resonance (1H-NMR) spectrometer was used to detect and quantify 41 metabolites; 21 (51.2%) of these were amino acids (Table S1). An analysis of similarity (ANOSIM) showed that the metabolic profiles were significantly different in the CBD and non-CBD stone groups (*p* = 0.0017) (Figure 4). Acetate and formate were signature metabolites and more abundant in the CBD stone group, while asparagine levels were significantly higher in the bile from the non-CBD stone patients (Figure 5).

**Figure 6:**
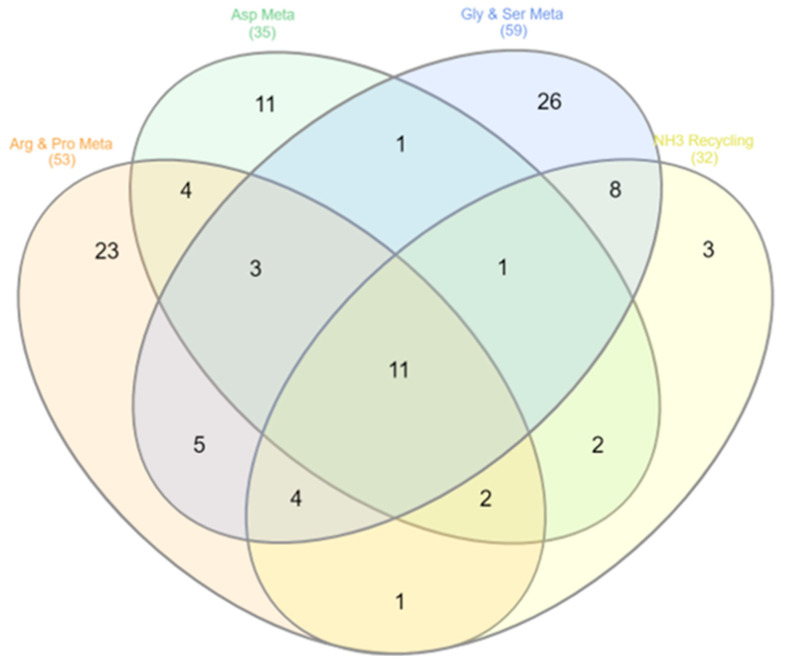
Pathway enrichment analysis was performed using MetaboAnalyst.

Additionally, a pathway enrichment analysis was performed using MetaboAnalyst to identify the pathways associated with the differential metabolites. Four metabolic pathways (Arginine and Proline Metabolism, Aspartate Metabolism, Glycine and Serine Metabolism, and Ammonia Recycling) were considered most likely to have caused metabolite changes. The pathway enrichment results were visualized using a Venn diagram (Figure 6). Furthermore, a functional enrichment analysis identified ammonia metabolism as an enriched term among the dysregulated metabolites (Figure S3).

### 3.5. Preclinical Experiment

**Figure 7:**
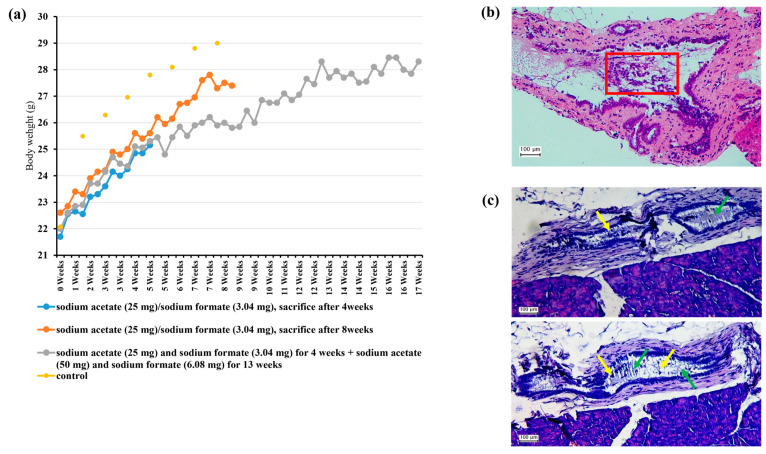
Preclinical Experiment. (**a**) Trends in the body weights of mice at 4, 8, and 17 weeks. (**b**) Bile duct with numerous intraepithelial neutrophils at 17 weeks. The densely populated and disorganized epithelial layer is infiltrated by numerous neutrophils (red box), indicative of acute inflammation. (**c**) The formation of neutrophil extracellular traps (NET, green arrows) and immune cell infiltration (myeloid cells, yellow arrows) through extracellular trap formation at 17 weeks.

A preclinical study was conducted to investigate the effects of acetate and formate, metabolites that are highly abundant in CBD stones, on the development of choledocholithiasis. We utilized a mouse model to administer sodium acetate (600 mM) and sodium formate (89.6 mM) intraperitoneally to 8-week-old C57BL/6 mice. Post-administration, the liver and bile ducts of these mice were extracted at intervals of 4, 8, and 17 weeks. No significant differences were observed between the control group and the experimental groups, whether in terms of the animal weights or the histopathological analysis findings at the initial 4-week stage. However, severe edematous changes in the small bowel, indicative of systemic inflammation, were observed in the treatment groups at the 8-week and 17-week stages. Remarkably, a microscopic exam noted the formation of extracellular traps and net structures. Moreover, immune cell infiltration through these extracellular traps was observed via the bile duct (Figure 7a–c).

## 4. Discussion

Our main hypothesis was that the composition of the bile microbiome is altered in patients with CBD stone disease and that an increased exposure to this altered microbiome may be involved in the development of choledocholithiasis. In the present study, we identified dysbiosis and the potentially relevant metabolites in the bile microbiomes of CBD stone patients and investigated the metabolic pathways associated with these metabolites. The results obtained showed that the levels of these metabolites were significantly higher in the bile of CBD stone patients. Notably, the abundance of *Enterococcus* in the bile microbiome of the CBD stone patients was significantly greater than that in the non-CBD stone group. In the CBD stone group, the *Bacillota* (formerly *Firmicutes*) mainly consisted of *Enterococcus*, however, in the non-CBD stone group, other *Bacillota* (formerly *Firmicutes*) mainly consisted of others. These results are consistent with those of our previous report [20]. Furthermore, the acetate and formate levels were significantly higher in the CBD stone group.

*Enterococcus* species are large genus of the phylum *Bacillota* (formerly *Firmicutes*) and have been well-documented for their ability to metabolize citrate [21,22], which, in *Enterococcus*, involves the transport of citrate across the cell membrane and its subsequent cleavage into acetyl-CoA and oxaloacetate by citrate lyase [22]. Consequently, *Enterococcus* produces acetate and formate. This suggests that the significant increases in acetate and formate in the CBD stones group found in our study were statistically strongly correlated with the increased *Enterococcus* in CBD stones [21,23]. Acetate plays a crucial role in the regulation of host metabolism and immunity, and reports have linked it to inflammation [24,25]. Several studies have shown that acetate can exacerbate inflammation and promote weight loss, which is consistent with our preclinical study results. While the involvement of formate in inflammation is not as well researched as that of acetate, some studies have suggested that formate could inhibit the differentiation and functions of dendritic cells, which are essential for immune responses [26]. Additionally, recent research has found that formate produced by gut Enterobacteriaceae can induce an inflammatory response in the colon and lead to colitis in mice [27]. Based on these findings, we postulate that the elevated abundance of *Enterococcus* in the bile microbiome of the CBD stone patients potentially contributed to the biosynthesis of acetate and formate.

Our study aimed not only to descriptively analyze microbiomates and metabolites, but also to see how they influence the pathogenicity of choledocholithiasis. Our current study found that acetate and formate, besides being metabolic products of Enterococcus, also play important roles in inflammation and potentially impact the pathogenesis and progression of CBD stone disease. Our preclinical experiments indicated that acetate and formate might induce neutrophil extracellular trap (NET) formation, which suggests a link between these metabolites and the development of inflammation, specifically through the involvement of NETs. The link between NETs and choledocholithiasis has recently been increasingly investigated, including by Munoz et al., who proposed that NETs promote the aggregation of biliary cholesterol and calcium crystals and gallstone formation [28]. Our findings are consistent with this proposal. Although the mechanism of stone formation was not elucidated by our mouse model study, we observed a significant increase in the presence of NETs in the biliary tract following the administration of acetate or formate to the mice and resultant severe systemic inflammation. Our results suggest that NET formation triggered by acetate or formate contributes to the pathogenesis of CBD stone disease. These results provide important insights into the complex interplay between the bile microbiome, metabolites, and immune system in biliary diseases. However, further investigations are needed to elucidate the involvement of NETs in the development of CBD stone disease and the mechanisms responsible, and to determine the effects of acetate and formate on NET formation and their subsequent impacts on inflammation.

Our findings concur with those reported by Zhang et al. in regard to a significant decrease in microbiome diversity and the abundance of certain phylogroups, particularly *Bacillota* (formerly *Firmicutes*) (*p* < 0.05), the largest phylum represented in the gut microbiota of the CBD stone patients [17]. This agreement is particularly intriguing, as Zhang et al. analyzed the gut microbiome and not the bile microbiome. Future investigations are required to examine the correlation between changes in the bile and gut microbiomes in CBD stone patients. The present study offers an innovative perspective that expands the understanding of the bile microbiota and highlights the significance of the complex relationship between the gut and bile microbiomes in the background of CBD stone disease.

Although our study sheds light on the bile microbiome and its associated metabolites in patients with CBD stones, several limitations need to be acknowledged. First, the sample size in our study was relatively small, which limits the generalizability of our findings to larger populations. Second, we focused solely on patients with CBD stones, and therefore, our results cannot be applied to other biliary diseases. Third, although we conducted preclinical experiments to investigate the mechanism responsible for acetate and formate, further studies are required to confirm our findings. We suggest that the adoption of multi-omics analyses in future studies would provide a more comprehensive understanding of the bile microbiome. Finally, we analyzed the microbiomes and metabolites at a single time point, and thus, dynamic changes in the bile microbiome and metabolites remain unclear. Future longitudinal studies are needed to investigate changes in the bile microbiome and metabolites over time and their implications for the progression of biliary diseases.

In conclusion, our findings demonstrate that the relationship between microbiota dysbiosis and gallstone formation needs to be further explored, which holds great significance for understanding the mechanism of gallstone formation.

## Figures and Tables

**Table 1:** Demographic characteristics of the study subjects.

Variables	Total (n = 25)	CBD Stones (n = 17)	non-CBD Stones (n = 8)	* *p*-Value
Age (years) ^§^	72 (42–89)	72 (43–89)	71 (42–81)	0.54
Sex, male, n (%)	13 (52.0)	8 (47.1)	5 (62.5)	0.49
Hypertension, n (%)	14 (56.0)	8 (47.1)	6 (75.0)	0.20
DM, n (%)	7 (28.0)	5 (29.4)	2 (25.0)	0.83
Dyslipidemia, n (%)	10 (40.0)	7 (41.2)	3 (37.5)	0.87
WBC (/ul) ^§^	8976 (3710–21,650)	8577 (3710–18,480)	9824 (5410–21,650)	0.56
CRP, mg/dL ^§^	4.8 (0.1–22.4)	6.5 (0.1–22.4)	1.2 (0.1–5.0)	0.06
T.bil, mg/dL ^§^	4.8 (0.1–22.4)	2.2 (0.3–8.7)	3.9 (0.2–23.9)	0.45
AST, IU/L ^§^	2.7 (0.2–23.9)	180.4 (12.0–920.0)	51.5 (19.0–130.0)	0.16
ALT, IU/L ^§^	139.1 (12.0–920.0)	145.9 (8.0–510.0)	36.6 (10.0–89.0)	0.07
ALP, IU/L ^§^	110.9 (8.0–510.0)	183.4 (41.0–646.0)	93.3 (45.0–153.0)	0.14

WBC, white blood cell count; CRP, C-reactive protein; T.bil, total bilirubin; AST, alanine aspartate transferase; ALT, alanine aminotransferase; and ALP, alkaline phosphatase; ^§^, median (range); * *p* values were calculated using the *t*-test or the Chi-square test.

## Data Availability

The raw data are available from the corresponding author on receipt of reasonable request and have been deposited in the publicly accessible NCBI database.

## Supplementary Materials

The following supporting information can be downloaded at: https://www.mdpi.com/article/10.3390/biomedicines11082124/s1. Table S1: A comprehensive enumeration of the forty-one metabolites identified using 1H-NMR spectrometer; Figure S1: There were no significant variations in microbiome composition based on age; Figure S2: Microbiome compositions at the major phylum and genus levels; Figure S3: Functional enrichment analysis.

## Abbreviations

CBD, common bile duct; ERCP, endoscopic retrograde cholangiopancreatography; ENBD, endoscopic nasobiliary drainage; PCR, polymerase chain reaction; NMR, nuclear magnetic resonance; LEfSe, linear discriminant analysis effect sizes; LDA, linear discriminant analysis; SCFA, short-chain fatty acid; and NET, neutrophil extracellular trap.
