# Supplementary material for: The Role of the Bile Microbiome in Common Bile Duct Stone Development
Source: Biomedicines. 2023 Jul 27;11(8):2124. doi: 10.3390/biomedicines11082124 (PMC10452286; doi:10.3390/biomedicines11082124)
Supplement: Supplementary file 1 [file biomedicines-11-02124-s001.zip › Table S1.pdf]

Table S1. A comprehensive enumeration of the forty-one metabolites identified using 1H-NMR spectrometer.

2-Hydroxybutyrate	Citrate	Glycocholate	Ornithine
3-Hydroxybutyrate	Creatine	Histidine	Phenylalanine
Acetate	Creatine phosphate	Isobutyrate	Proline
Acetoacetate	Erythritol	Isoleucine	Serine
Alanine	Formate	Lactate	Succinate
Asparagine	Glucose	Leucine	Taurodeoxycholic acid
Aspartate	Glucuronate	Lysine	Threonine
Chenodeoxycholic acid	Glutamate	Methanol	Tryptophan
Cholate	Glutamine	Methionine	Tyrosine
Choline	Glycine	O-Phosphocholine	Valine
sn-Glycero-3-phosphocholine			
