# Supplementary figures and images for: The Role of the Bile Microbiome in Common Bile Duct Stone Development
Source: Biomedicines. 2023 Jul 27;11(8):2124. doi: 10.3390/biomedicines11082124 (PMC10452286; doi:10.3390/biomedicines11082124)

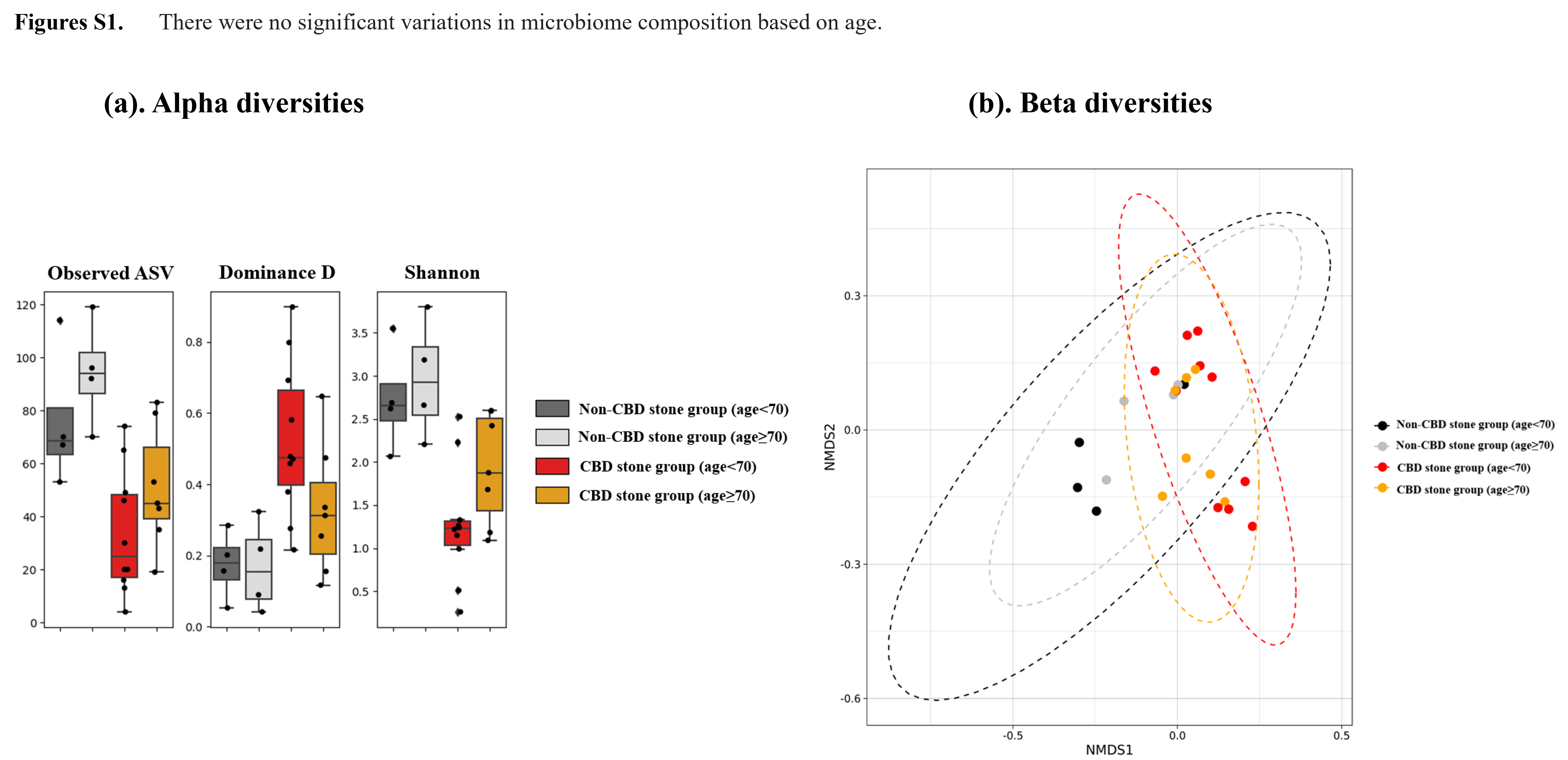

Supplement: Supplementary file 1 [file biomedicines-11-02124-s001.zip › FigureS1a and S1b.png]

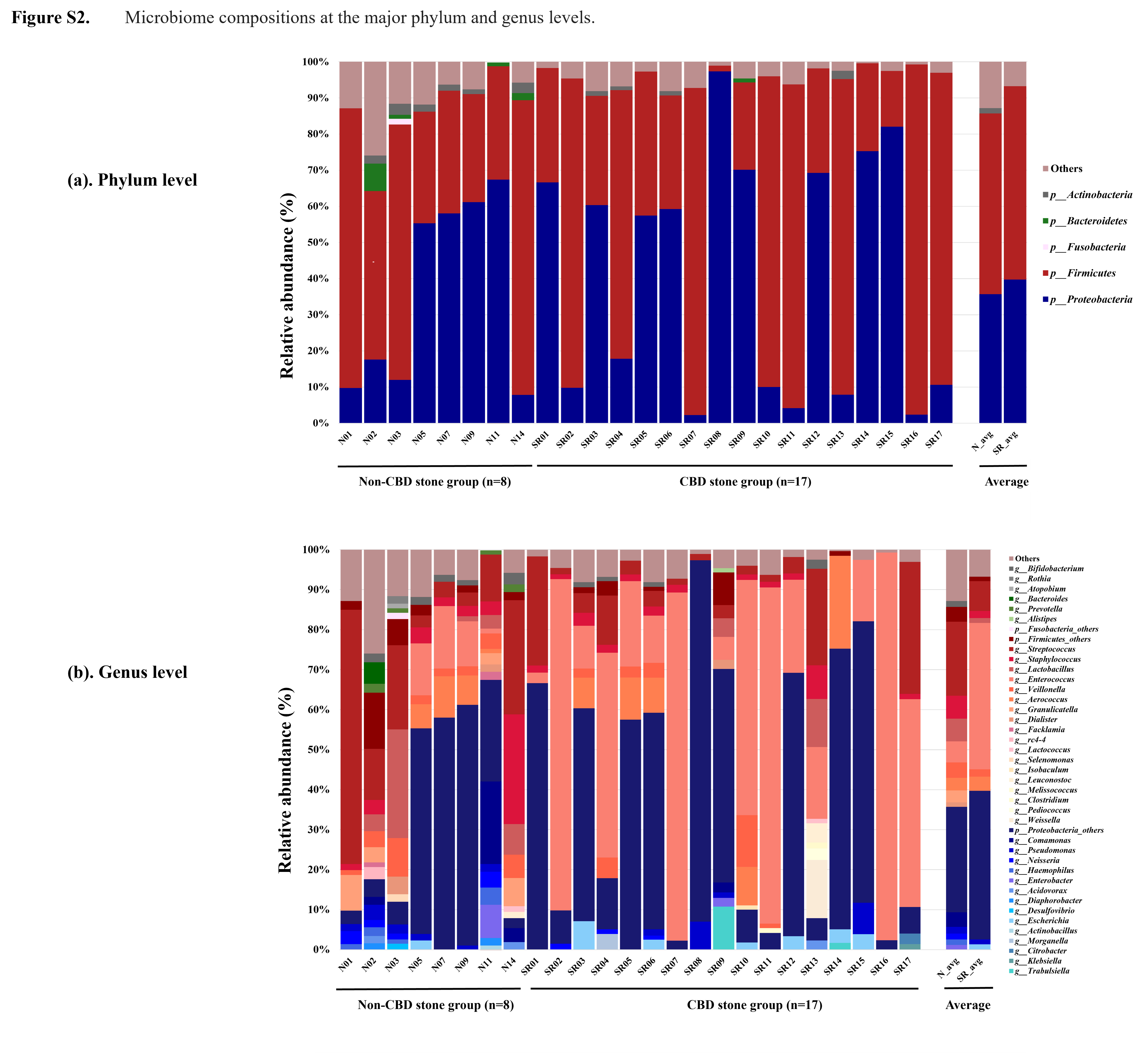

Supplement: Supplementary file 1 [file biomedicines-11-02124-s001.zip › FigureS2a and S2b_0803.png]

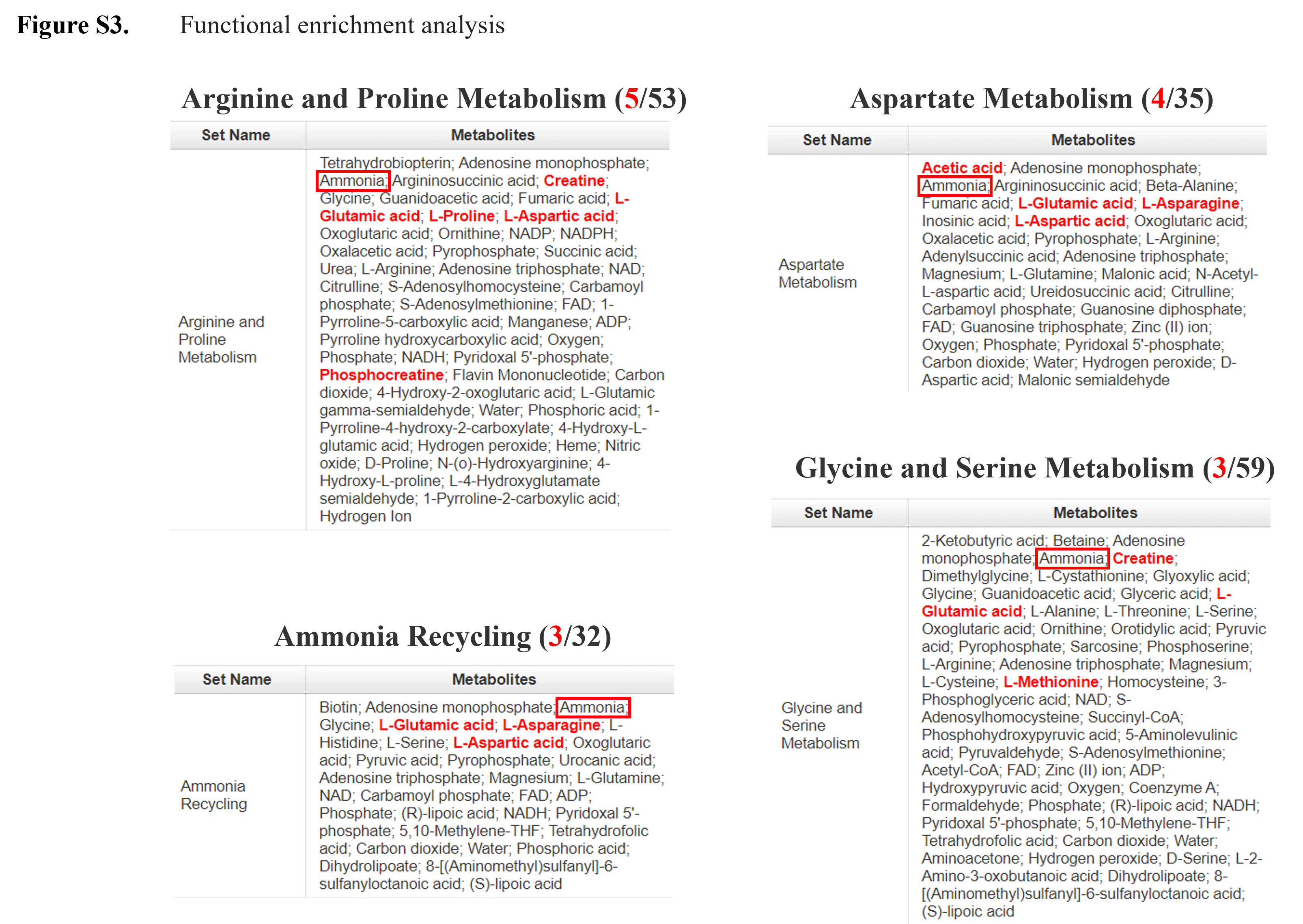

Supplement: Supplementary file 1 [file biomedicines-11-02124-s001.zip › FigureS3_0717.png]
